# Supplementary material for: Distinct spatial immune microlandscapes are independently associated with outcomes in triple-negative breast cancer
Source: Nat Commun. 2023 Apr 18;14:2215. doi: 10.1038/s41467-023-37806-0 (PMC10113250; doi:10.1038/s41467-023-37806-0)
Supplement: Supplementary file 1 — Supplementary Information [file 41467_2023_37806_MOESM1_ESM.pdf]

	RFS-NO (N=22)	RFS-YES (N=22)	Total (N=44)	p value
**Pathologic Tumor Stage**				0.348
1	6 (27.3%)	7 (31.8%)	13 (29.5%)	
2	14 (63.6%)	15 (68.2%)	29 (65.9%)	
3	2 (9.1%)	0 (0.0%)	2 (4.5%)	
**Pathologic Nodal Stage**				0.233
0	4 (18.2%)	5 (22.7%)	9 (20.5%)	
1	10 (45.5%)	14 (63.6%)	24 (54.5%)	
2	5 (22.7%)	3 (13.6%)	8 (18.2%)	
3	3 (13.6%)	0 (0.0%)	3 (6.8%)	
**Age (years)**				0.417
- Median (Range)	53.5 (35.0, 65.0)	52.0 (38.0, 65.0)	53.0 (35.0, 65.0)	
**RFS (years)**				< 0.001
- Median (Range)	2.3 (0.2, 8.3)	10.7 (9.0, 11.8)	8.7 (0.2, 11.8)	
**Tumor Size (mm)**				0.212
- Median (Range)	25.0 (10.0, 60.0)	22.5 (12.0, 50.0)	24.5 (10.0, 60.0)	

Supplementary Table 1: Clinical/pathological features of FinXX TNBC. DSP analysis was carried out on 44 samples from the FinXX TNBC cohort, 22 patients that recurred (RFS=NO) and 22 that did not (RFS=YES) during the trial. Half of the patients in each arm were selected from the capecitabine arm and half from the 5-FU arm of the trial. Samples were matched for age at diagnosis, tumor size, nodal status, and tumor stage. Chi-squared analysis was used to calculate two-sided p-values for tumor stage and node status. The Wilcoxon test was used to calculate two-sided p-values for age, RFS, and tumor size.

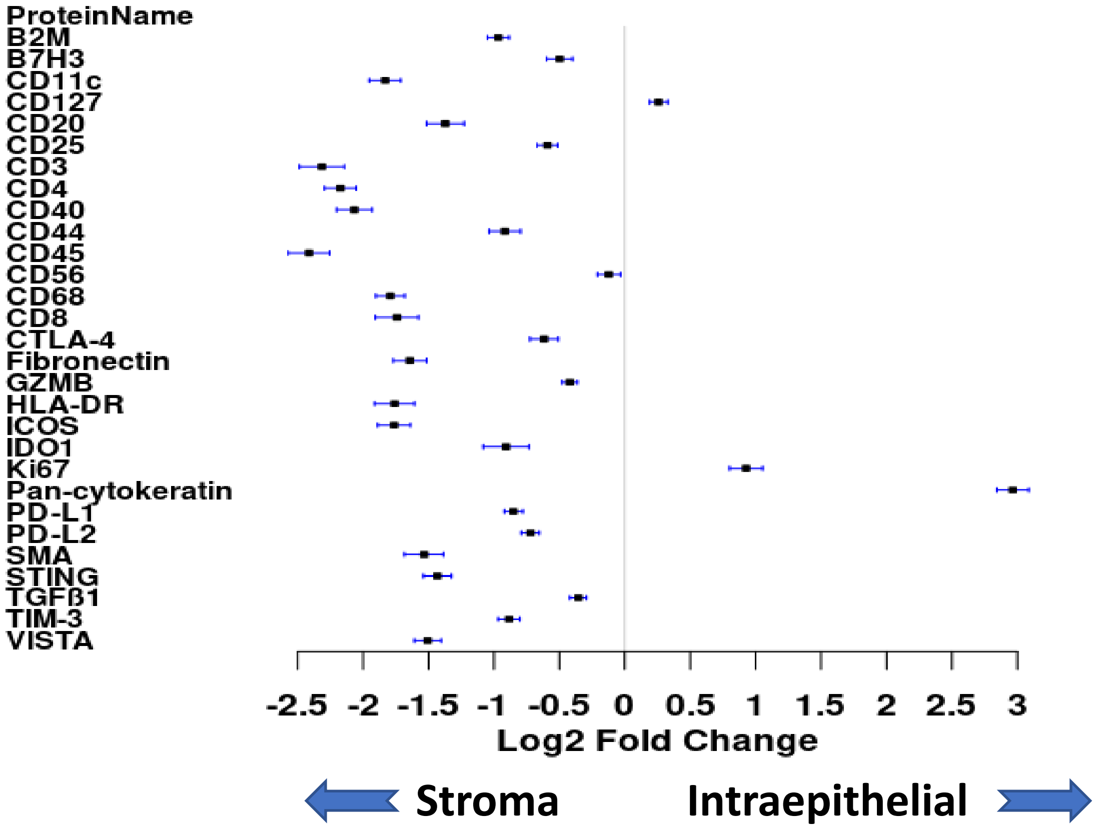

Supplementary Figure 1: Segment-level spatial distribution of immune proteins in FinXX TNBC. Segment level analysis for normalized protein counts within intraepithelial (All Intraepithelial, n=12) and stroma (All Stroma, n=8) segments for each tumor sample. The linear mixed model was used to estimate fold change, given as log2 Fold Change. Proteins that were more abundant in intraepithelial segments (CD127, pan-cytokeratin, and Ki-67) exhibit log2 Fold Change >0. Proteins that were more abundant in stroma segments exhibit log2 Fold Change <0. Whisker bars represent 95% confidence intervals for mean estimated log2 fold change, indicated by the black square. All proteins except CD56 were significantly differentially expressed in either intraepithelial or stroma segments at nominal $p<0.05$. Quantitative data are in Supplementary Data 6 and Source Data are in Supplementary Data 1. B2M: beta-2 microglobulin; GZMB: granzyme B; SMA: smooth muscle actin.

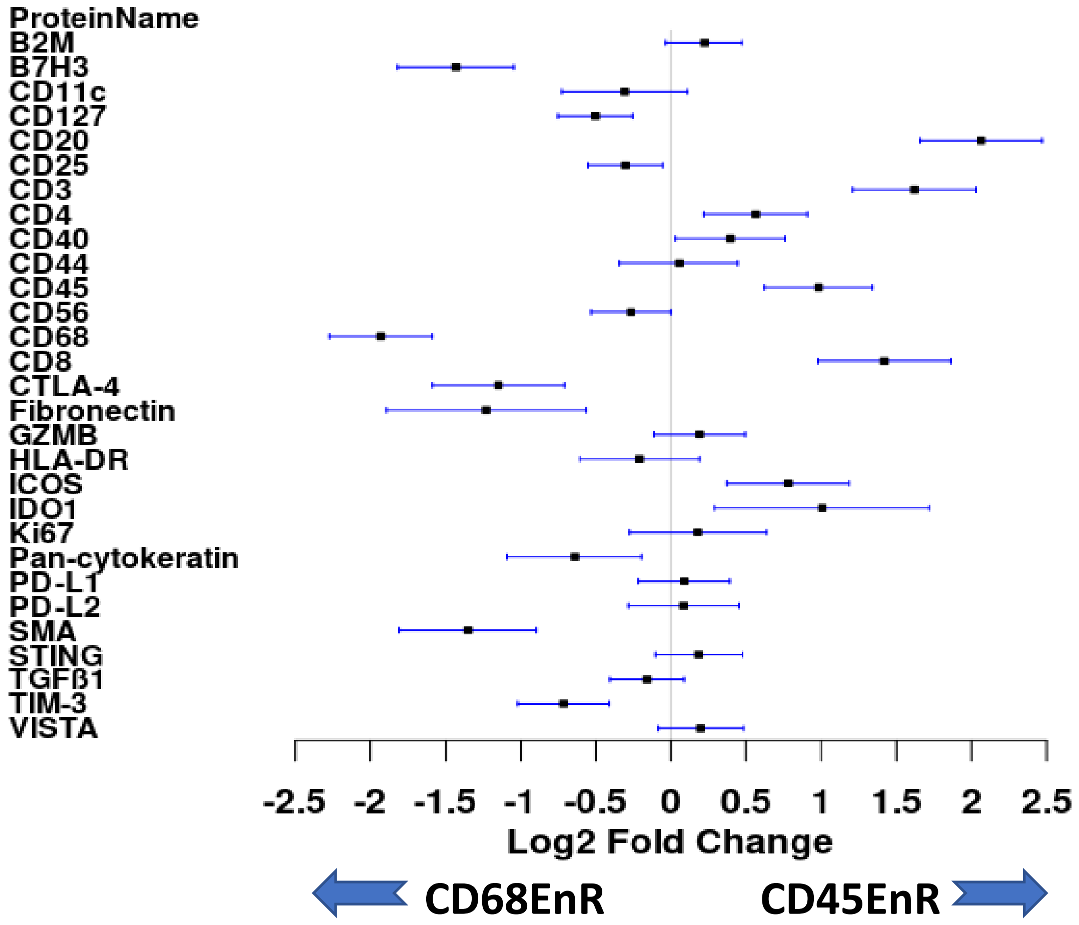

Supplementary Figure 2: Differential expression (log2 Fold Change) in CD45EnR stroma vs CD68EnR stroma segments in FinXX TNBC. Segment level counts (4 replicates of each segment per tumor) were averaged and the generalized linear model with negative binomial distribution was used to estimate fold change (log 2 Fold Change) for CD45EnR stroma vs CD68EnR stroma. Whisker bars indicate 95% confidence intervals about the mean, indicated by the black square. Nominal p-values were <0.05 for all comparisons in which 95% confidence intervals did not cross the reference line at log2 Fold Change = 0. Quantitative data are in Supplementary Data 6 and Source Data are in Supplementary Data 1. B2M: beta-2 microglobulin; GZMB: granzyme B; SMA: smooth muscle actin.

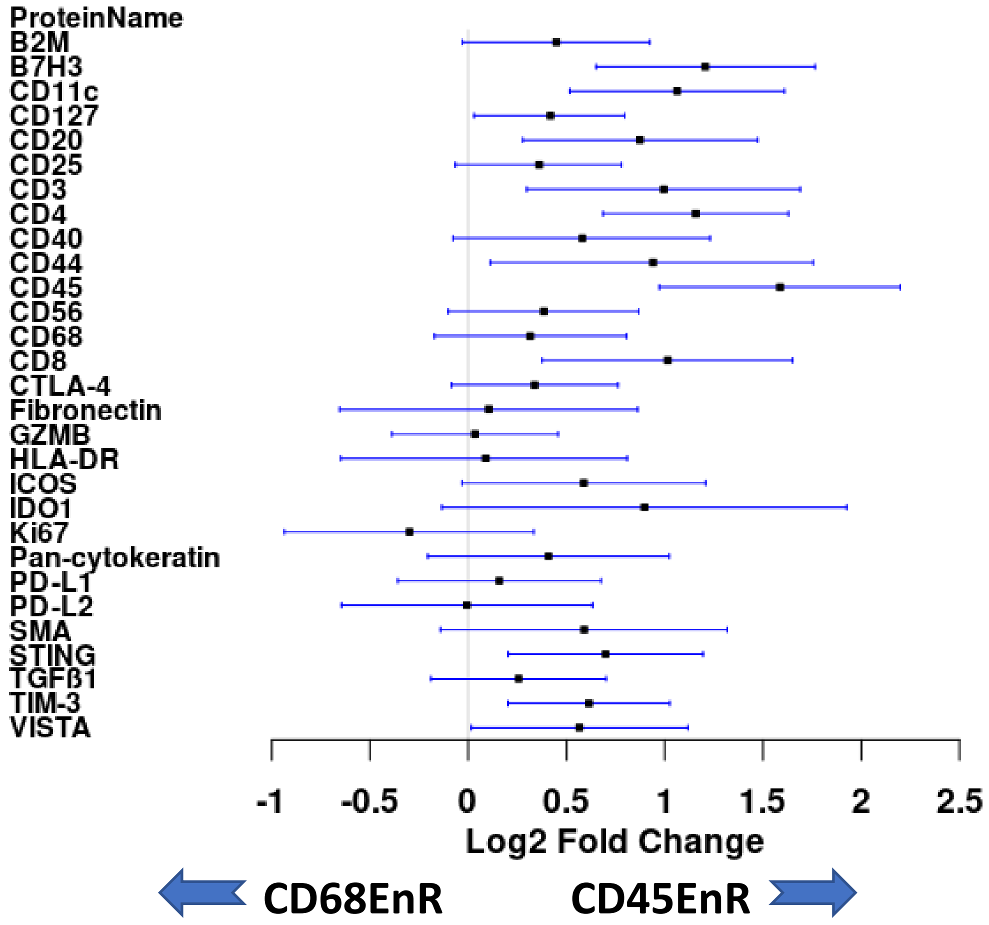

Supplementary Figure 3: Differential expression (log2 fold change) CD45EnR intraepithelial vs CD68EnR intraepithelial segments in FinXX TNBC. CD45EnR intraepithelial vs CD68EnR intraepithelial abundance was averaged (4 segments/tumor). Whisker lines indicate 95% confidence intervals about the mean, indicated by the black square. Nominal p-values were <0.05 for all comparisons in which 95% confidence intervals did not cross the reference line at log2 Fold Change = 0. Quantitative data are given in Supplementary Data 6 and Source Data are in Supplementary Data 1. B2M: beta-2 microglobulin; GZMB: granzyme B; SMA: smooth muscle actin.

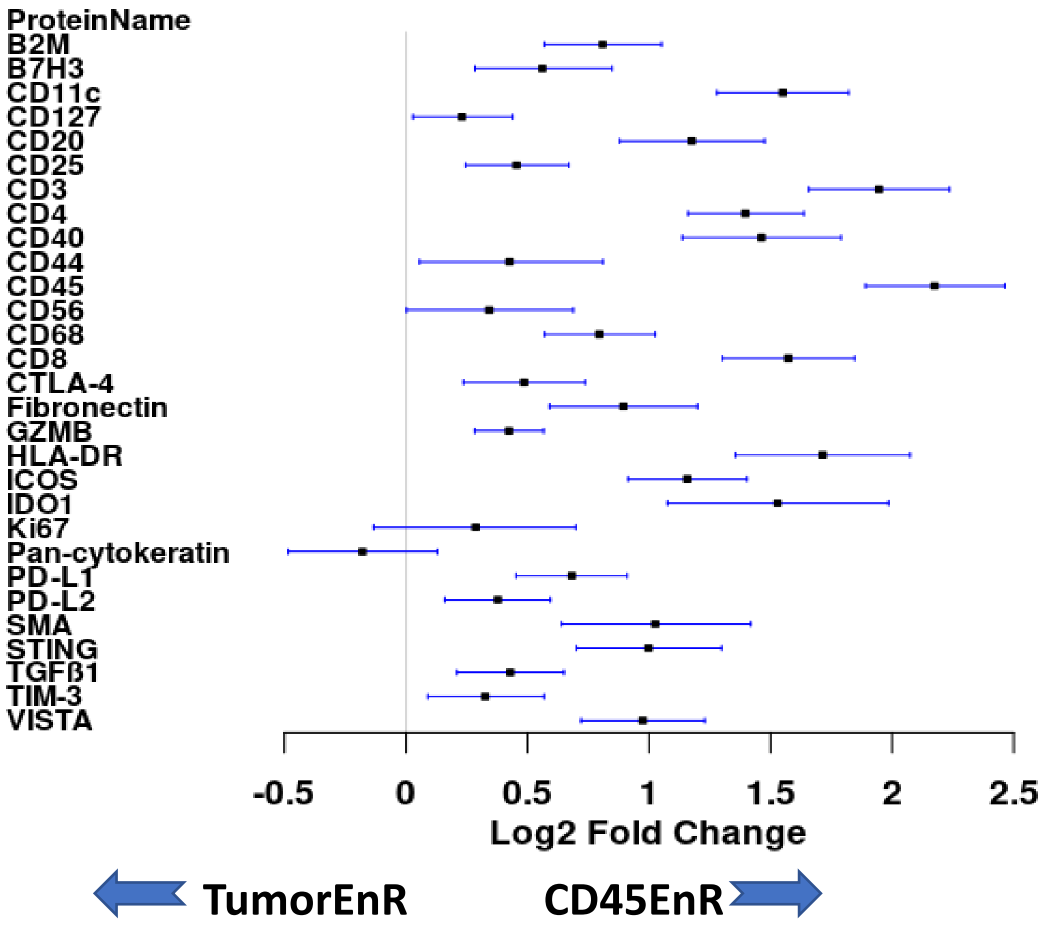

Supplementary Figure 4: Differential expression (log2 fold change) CD45EnR intraepithelial vs TumorEnR intraepithelial segments in FinXX TNBC. CD45EnR intraepithelial (n=4 replicate segments/tumor) vs TumorEnR (n=4 replicate segments/tumor) intraepithelial segments. Whisker lines indicate 95% confidence intervals about the mean, indicated by the black square. Nominal p-values were <0.05 for all comparisons in which 95% confidence intervals did not cross the reference line at log2 Fold Change = 0. Quantitative data are given in Supplementary Data 6 and Source Data are in Supplementary Data 1. B2M: beta-2 microglobulin; GZMB: granzyme B; SMA: smooth muscle actin.

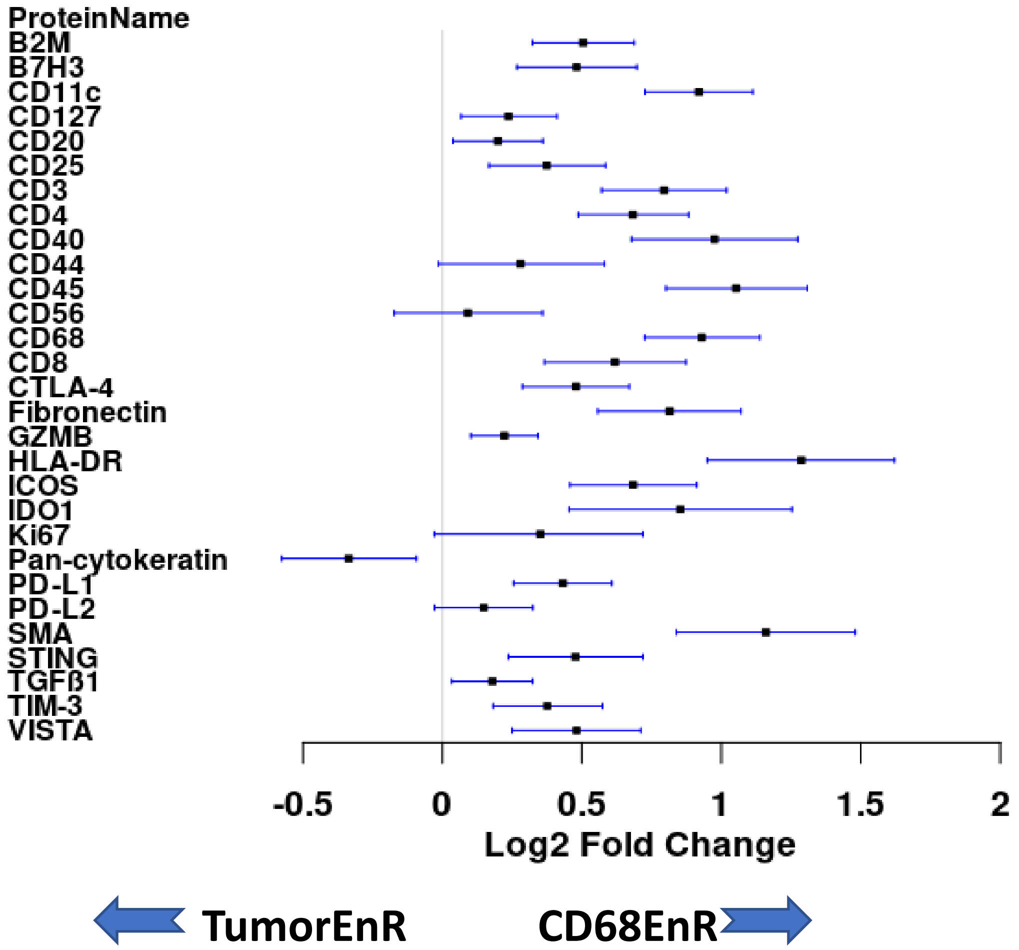

Supplementary Figure 5: Differential expression (log2 fold change) CD68EnR intraepithelial vs TumorEnR intraepithelial segments in FinXX TNBC. Four replicate segments were averaged for each tumor. Whisker lines indicate 95% confidence intervals about the mean, indicated by the black square. Nominal p-values were <0.05 for all comparisons in which 95% confidence intervals did not cross the reference line at log2 Fold Change = 0. Quantitative data are given in Supplementary Data 6 and Source Data are in Supplementary Data 1. B2M: beta-2 microglobulin; GZMB: granzyme B; SMA: smooth muscle actin.

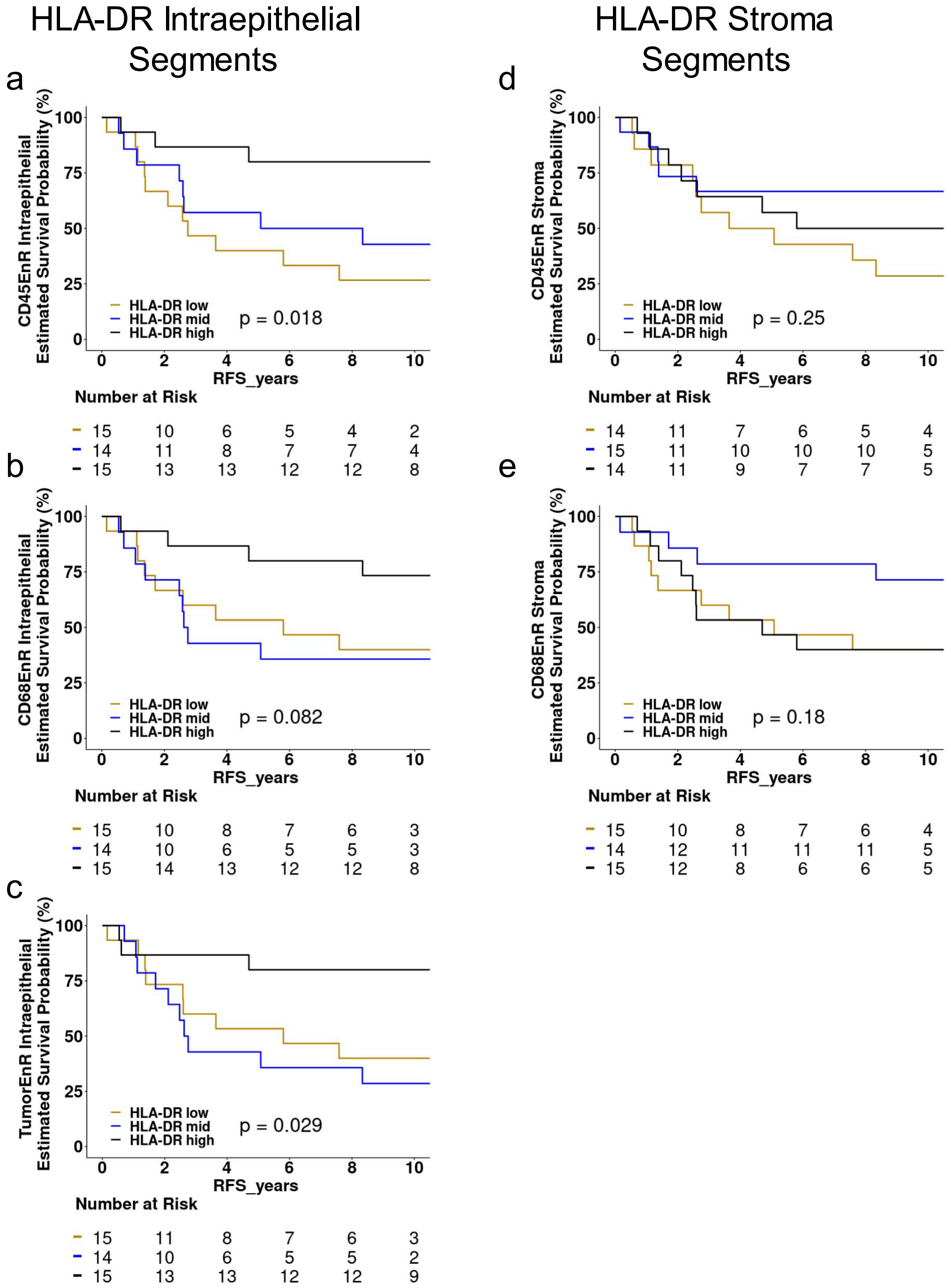

Supplementary Figure 6: Kaplan Meier Analysis of Recurrence-Free Survival as a Function of HLA-DR Protein Abundance in FinXX TNBC Segments. KM analysis was performed to assess the relationship between RFS and tumor average abundance of HLA-DR protein in CD45EnR (Panel a), CD68EnR (Panel b), and TumorEnR (Panel c) intraepithelial segments. Panels d and e show RFS as a function of stroma HLA-DR in CD45EnR and CD68EnR segments, respectively. HLA-DR protein abundance was sorted by tertiles (high=black line, mid=blue line, low=orange line). Log rank p-values for each analysis are given, as well as patients at risk for each time point in the analysis. Source data are in Supplementary Data 2.

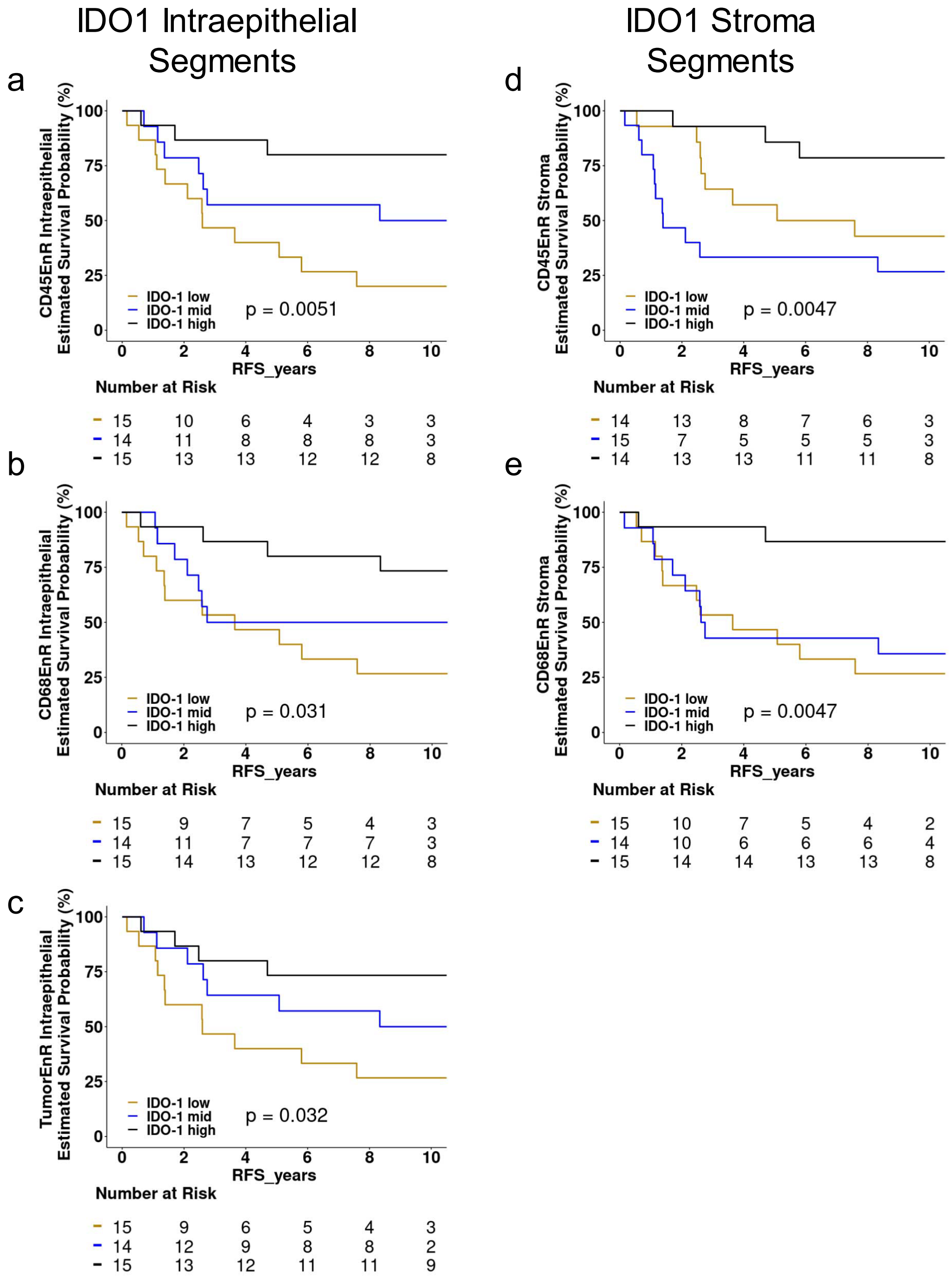

Supplementary Figure 7: Kaplan Meier Analysis of Recurrence-Free Survival as a Function of IDO1 protein abundance in FinXX TNBC Segments. KM analysis was performed to assess the relationship between RFS and tumor average abundance of IDO1 protein in CD45EnR (Panel a), CD68EnR (Panel b), and TumorEnR (Panel c) intraepithelial segments. Panels d and e show RFS as a function of stroma IDO1 protein in CD45EnR and CD68EnR segments, respectively. IDO1 abundance was sorted by tertiles (high=black line, mid=blue line, low=orange line). Log rank p-values for each analysis are given, as well as patients at risk for each time point in the analysis. Source data are in Supplementary Data 2.

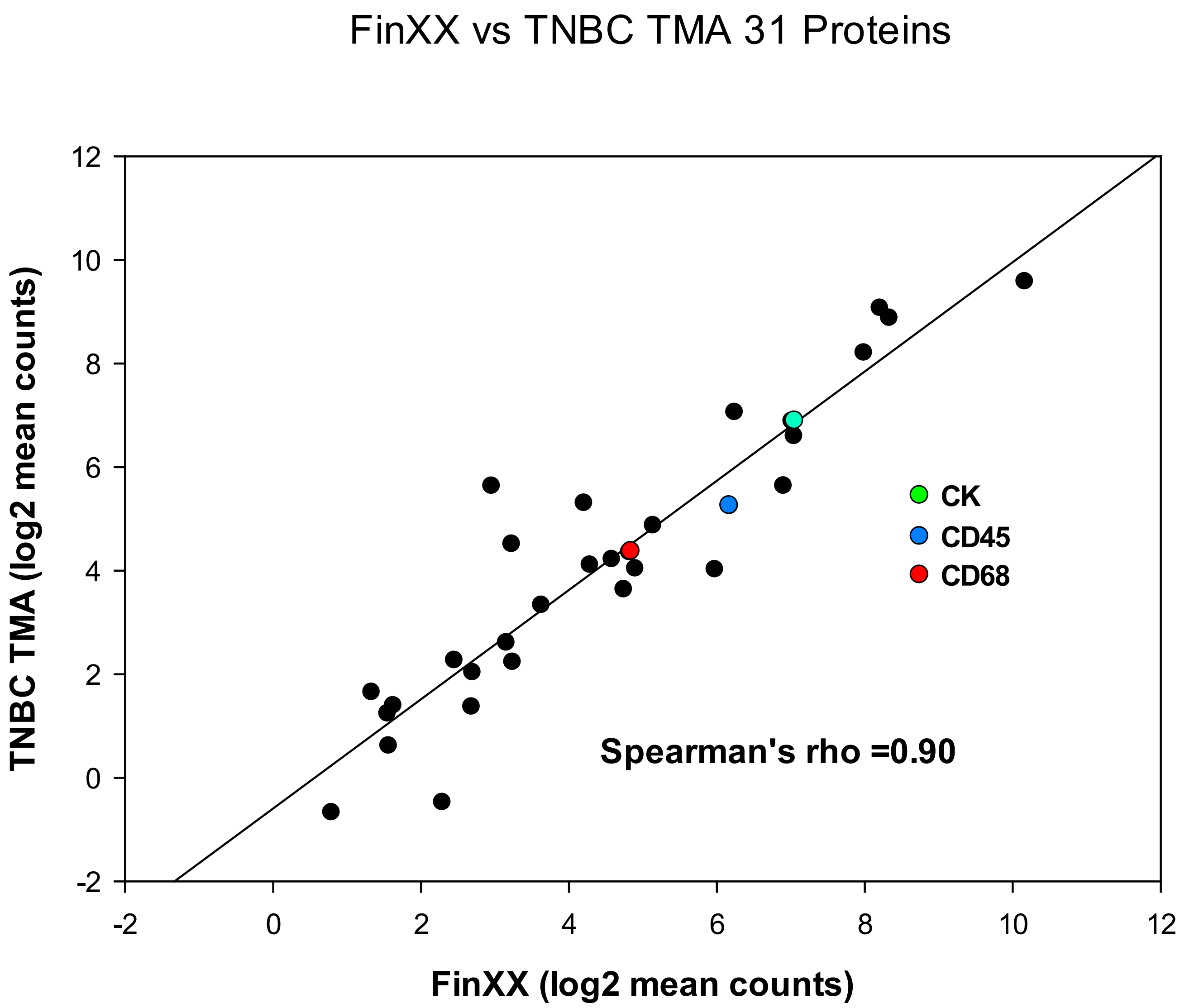

Supplementary Figure 8: Comparison of intraepithelial protein abundance in FinXX TNBC and Mayo Clinic TNBC TMA samples. Mean log2 counts for 31 protein targets common to both the FinXX and Mayo Clinic TMA DSP assays were calculated for each tumor. Data were averaged for the TNBC TMA and for all three intraepithelial segments from FinXX. source data for FinXX are in Supplementary Data 2 and Source Data for TNBC TMA are in Supplementary Data 7.

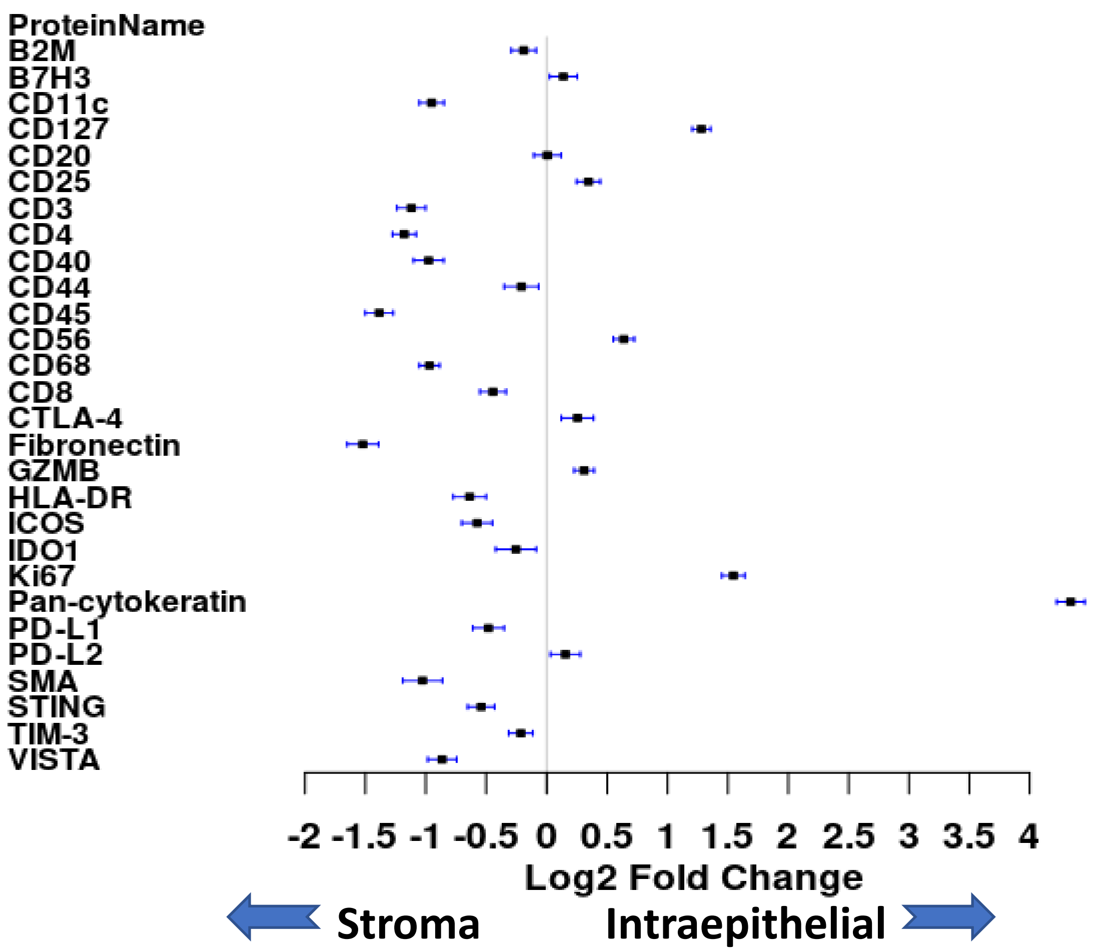

Supplementary Figure 9: Segment-level spatial distribution of immune proteins in Mayo Clinic TNBC TMA. The linear mixed model was used to estimate fold change (displayed as log2 fold change) comparing intraepithelial and stroma segments from TMA tumor cores. Whisker lines indicate 95% confidence intervals about the mean, indicated by the black dot. Nominal p-values were <0.05 for all comparisons in which 95% confidence intervals did not cross the reference line at log2 Fold Change = 0. Mean abundance is indicated by the black square. Source data are given in Supplementary Data 7. B2M: beta-2 microglobulin; GZMB: granzyme B; SMA: smooth muscle actin.

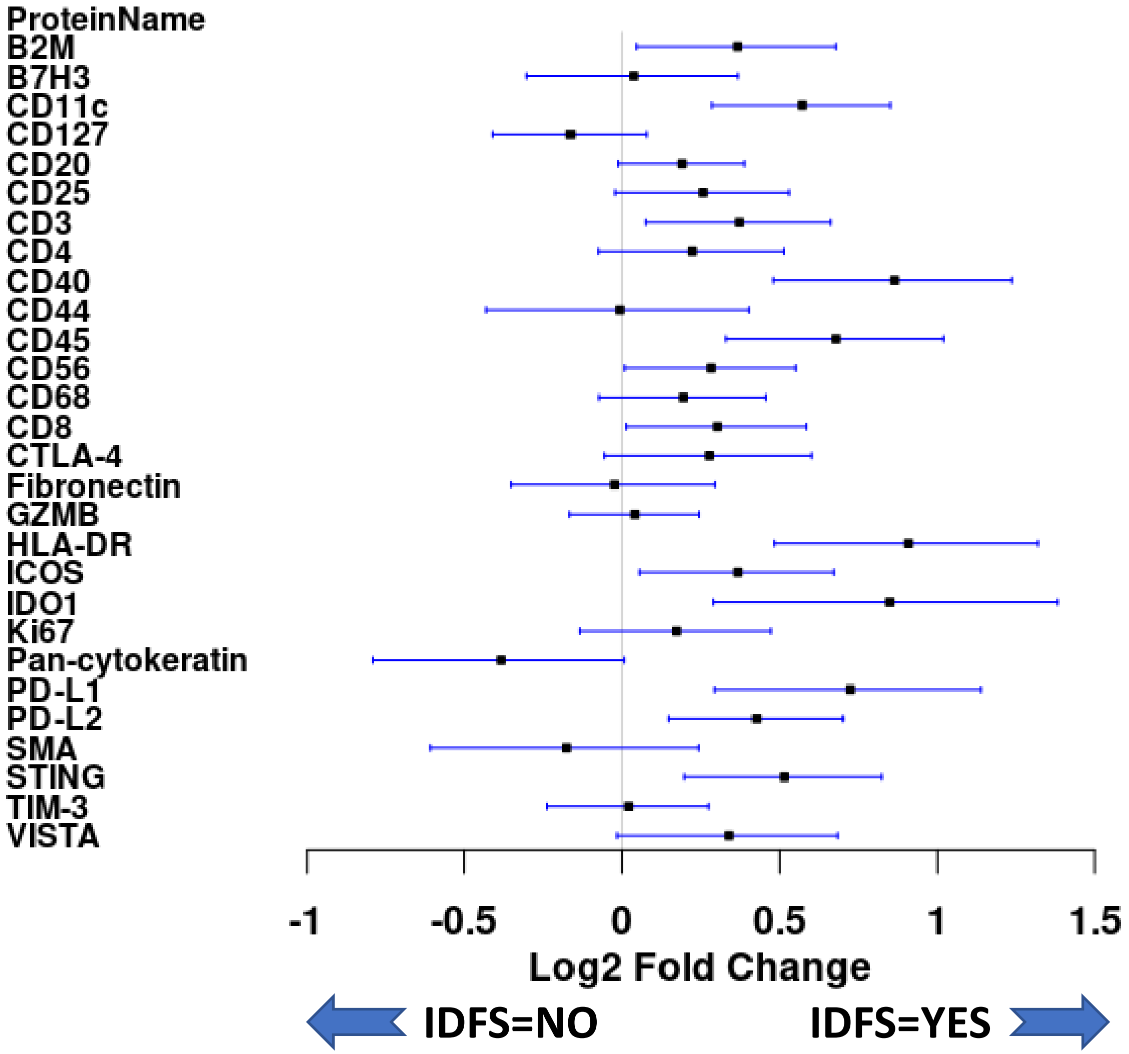

Supplementary Figure 10: Differential expression (log2 fold change) as a function of recurrence (RFS =YES vs. RFS=NO) in intraepithelial segments from the Mayo Clinic TNBC TMA. Differential expression of tumor average protein counts, comparing intraepithelial segments in tumors that recurred (RFS=NO) to those in tumors that did not recur (RFS=YES). Whisker bars indicate 95% confidence intervals for estimated fold change, given as log2 Fold Change. Proteins for which 95% confidence intervals did not cross the reference line at log2 Fold Change = 0 are significant at two-sided $p < 0.05$. Mean abundance is indicated by the black square. Source data are given in Supplementary Data 7. B2M: beta-2 microglobulin; GZMB: granzyme B; SMA: smooth muscle actin.

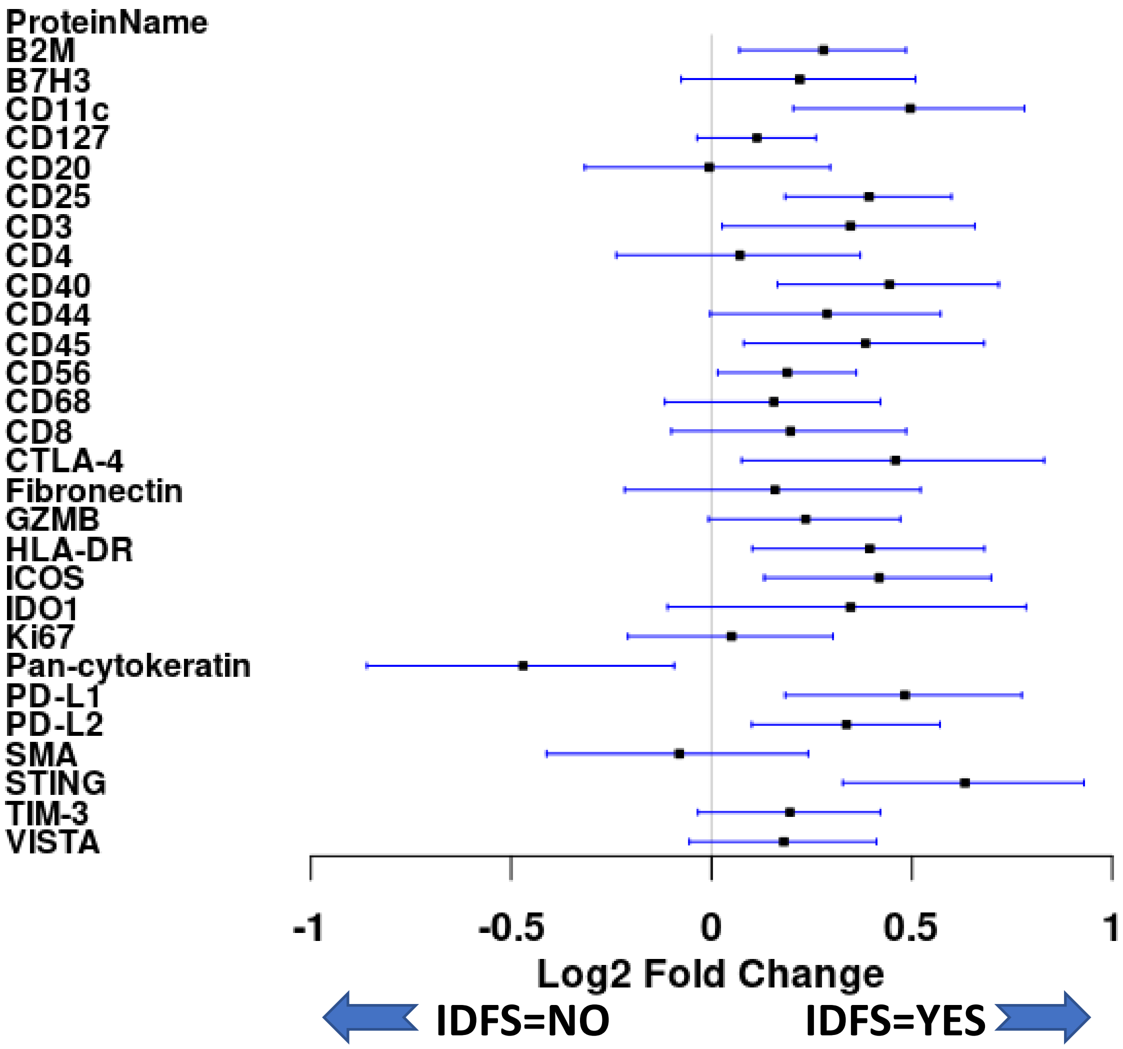

Supplementary Figure 11: Differential expression (log2 fold change) as a function of recurrence (RFS=YES vs. RFS=NO) in stromal segments from the Mayo Clinic TNBC TMA. Tumor average data were used to estimate differential expression, as log2 Fold Change in Stroma segments. Whisker bars indicate 95% confidence intervals for estimated fold change, given as log2 Fold Change. Proteins for which 95% confidence intervals did not cross the reference line at log2 Fold Change = 0 are significant at two-sided $p < 0.05$. Mean abundance is indicated by the black square. Source data are given in Supplementary Data 7. B2M: beta-2 microglobulin; GZMB: granzyme B; SMA: smooth muscle actin.

Protein	FinXX Estimated log2FC Intraepithelial Segments: RFS YES/NO	TNBC TMA Estimated log2FC Intraepithelial Segments: IDFS YES/NO
**B2M**	0.76	1.28
**CD11c**	0.53	1.51
**CD20**	1.05	1.13
**CD40**	0.98	1.7
**CD56**	1.61	1.18
**GZMB**	0.87	1.09
**HLA-DR**	1.49	1.98
**ICOS**	0.91	1.4
**IDO1**	0.90	2.13
**PD-L1**	0.78	1.7
**PD-L2**	1.96	1.21

Supplementary Table 2: Differential expression of selected immune proteins as a function of recurrence in intraepithelial segments from FinXX and Mayo Clinic TNBC cohorts. Differential expression, given as log2 transformed fold change (log2FC) was estimated from the linear mixed model. Differential expression is given for 10 proteins with BYadj $p<0.05$ plus PD-L1. Source data for FinXX are in Supplementary Data 1 and for TNBC_TMA in Supplementary Data 7. B2M: beta-2 microglobulin; GZMB: granzyme B.

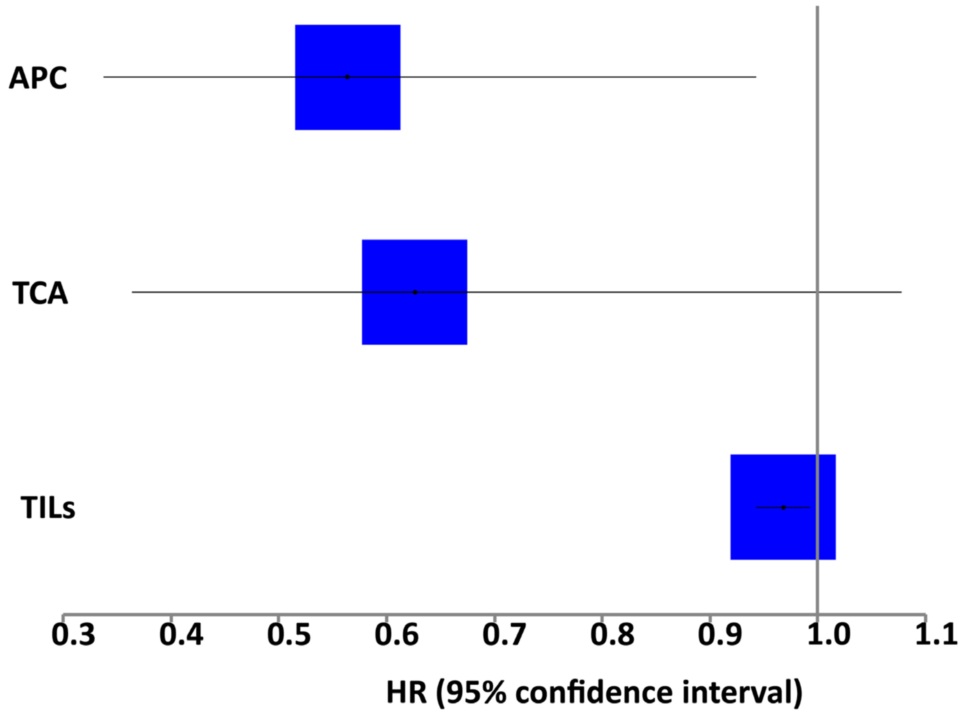

Supplementary Figure 12: Cox Hazard Ratios (HR) for FinXX samples. Cox HRs were calculated for the intraepithelial APC eigenprotein score, the intraepithelial TCA eigenprotein score, or for stromal TILs. Source data are in Supplementary Data 5.

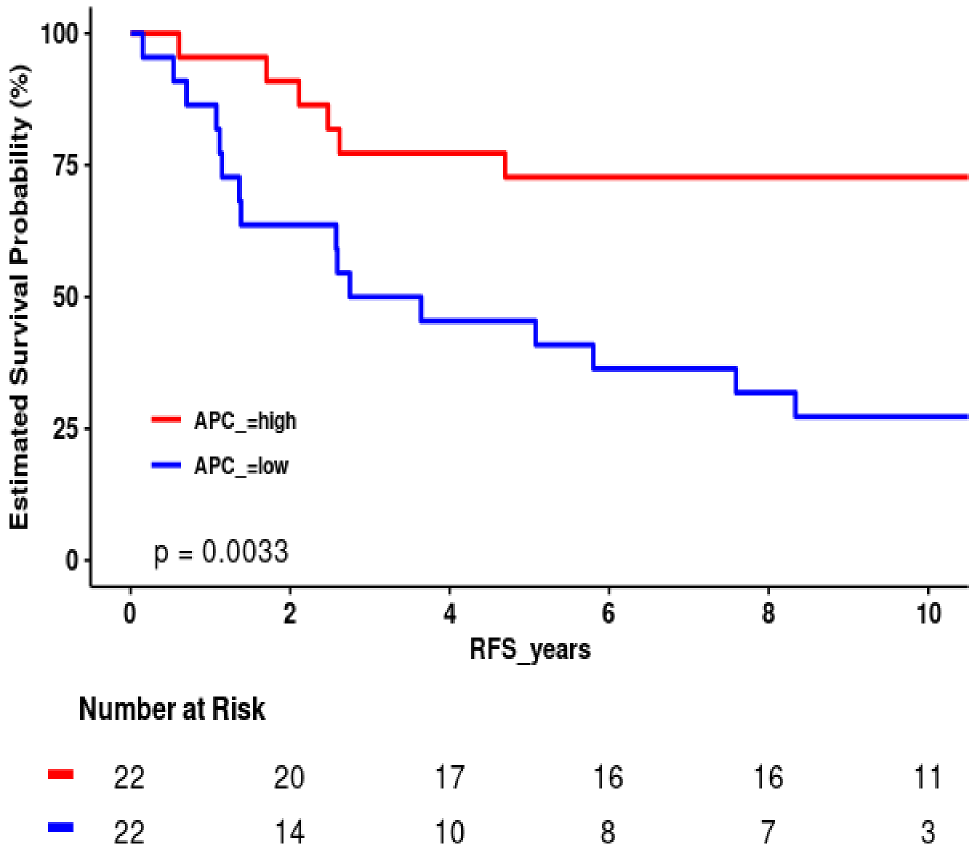

Supplementary Figure 13: Kaplan-Meier analysis of recurrence in FinXX samples by intraepithelial APC eigenprotein score. Log rank p-value is given for APC high (red line) vs APC low (blue line) with APC high defined as APC score >median. Source data are in Supplementary Data 5

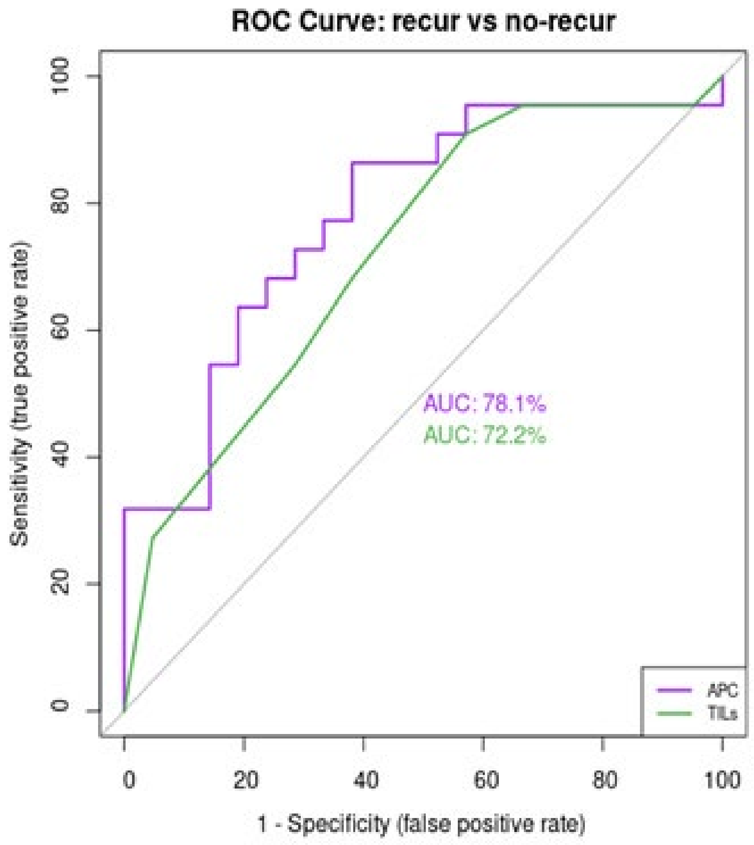

Predictor	threshold	sensitivity	specificity	ppv	npv	AUC
RFS_c_APC	-0.137	0.864	0.636	0.704	0.824	0.781
RFS_c_TILs	45.000	0.909	0.429	0.625	0.818	0.722

Supplementary Figure 14: ROC analysis to calculate the area under the curve (AUC) for the intraepithelial APC eigenprotein score (lavender) or stromal TILs (green) for FinXX samples. Patient numbers: 22 recurred and 22 did not recur. Source data are in Supplementary Data 5.

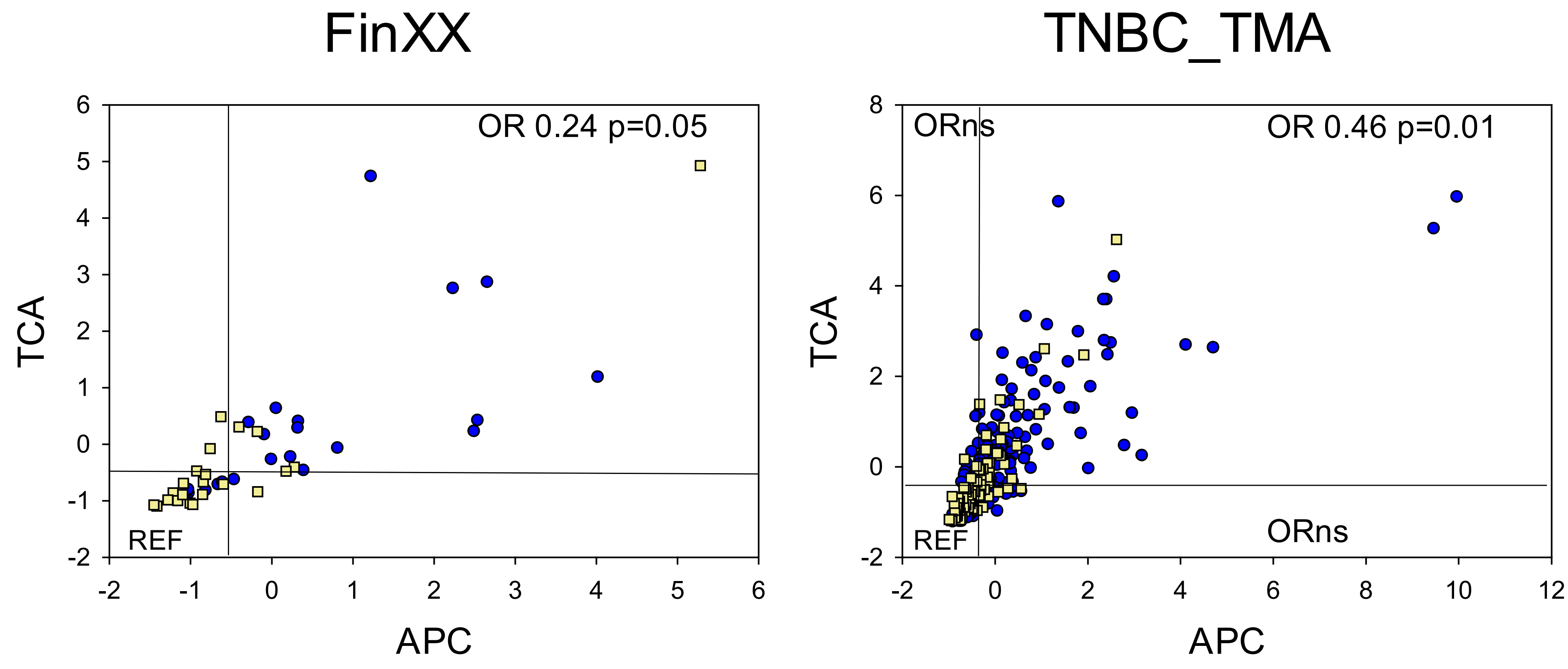

Supplementary Figure 15: Recurrence as a function of APC and TCA eigenprotein scores in FinXX and TNBC TMA samples. Each symbol represents an individual patient who recurred (yellow squares) or did not recur (blue circles) within the cohort irrespective of time of recurrence. Odds Ratios (OR) for five-year recurrence are shown if two-sided p-value was <0.05. ORns indicates p>0.05. Source data for eigenprotein scores are in Supplementary Data 5. ORs for all comparisons are given in Supplementary Table 3. Patient numbers: FinXX, 22 recurred and 22 did not recur; TNBC_TMA, 81 recurred and 181 did not recur.

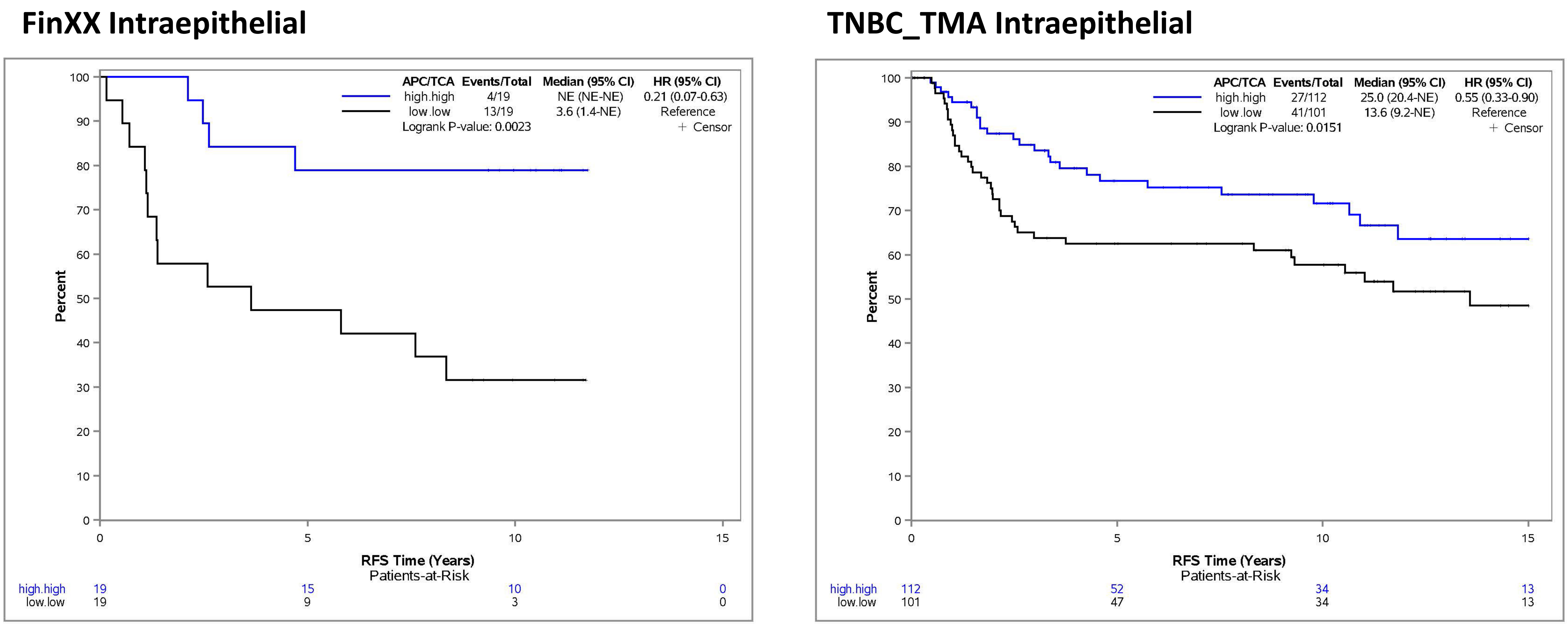

Supplementary Figure 16: Kaplan-Meier analysis comparing high vs. low APC and TCA eigenprotein scores in FinXX (left) and Mayo Clinic TNBC TMA samples (right). APC or TCA scores are defined as high if > median, with APC/TCA high (blue line) and APC/TCA low defined as < median for both scores (black line). Cox HRs, median survival, and log rank p-values are given in the insert. Source data are in in Supplementary Data 5.

Supplementary Table 3

FinXX Intraepithelial					
	No recur at 5yrs (N=26)	Recur at 5yrs (N=18)	Total (N=44)	OR.95CI	p.value
**APC.TCA.levels**					
high.high	15 (57.7%)	4 (22.2%)	19 (43.2%)	0.24(0.052,0.945)	**0.05**
high.low	1 (3.8%)	2 (11.1%)	3 (6.8%)	1.8(0.147,42.704)	0.653
low.high	1 (3.8%)	2 (11.1%)	3 (6.8%)	1.8(0.147,42.704)	0.653
low.low	9 (34.6%)	10 (55.6%)	19 (43.2%)	ref	ref
**APC.PD_L1**					
high.high	12 (46.2%)	4 (22.2%)	16 (36.4%)	0.2(0.04,0.861)	**0.04**
high.low	4 (15.4%)	2 (11.1%)	6 (13.6%)	0.3(0.033,2.029)	0.232
low.high	4 (15.4%)	2 (11.1%)	6 (13.6%)	0.3(0.033,2.029)	0.232
low.low	6 (23.1%)	10 (55.6%)	16 (36.4%)	ref	ref
**APC.IDO1**					
high.high	13 (50.0%)	4 (22.2%)	17 (38.6%)	0.168(0.034,0.704)	**0.02**
high.low	3 (11.5%)	2 (11.1%)	5 (11.4%)	0.364(0.039,2.782)	0.333
low.high	4 (15.4%)	1 (5.6%)	5 (11.4%)	0.136(0.006,1.182)	0.105
low.low	6 (23.1%)	11 (61.1%)	17 (38.6%)	ref	ref
**TCA.PD_L1**					
high.high	13 (50.0%)	4 (22.2%)	17 (38.6%)	0.215(0.044,0.895)	**0.04**
high.low	3 (11.5%)	2 (11.1%)	5 (11.4%)	0.467(0.051,3.539)	0.463
low.high	3 (11.5%)	2 (11.1%)	5 (11.4%)	0.467(0.051,3.539)	0.463
low.low	7 (26.9%)	10 (55.6%)	17 (38.6%)	ref	ref
**TCA.IDO1**					
high.high	12 (46.2%)	2 (11.1%)	14 (31.8%)	0.093(0.011,0.511)	**0.01**
high.low	4 (15.4%)	4 (22.2%)	8 (18.2%)	0.556(0.09,3.293)	0.514
low.high	5 (19.2%)	3 (16.7%)	8 (18.2%)	0.333(0.049,1.942)	0.232
low.low	5 (19.2%)	9 (50.0%)	14 (31.8%)	ref	ref

TNBC TMA Intraepithelial					
	No recur at 5yrs (N=201)	Recur at 5yrs (N=61)	Total (N=262)	OR.95CI	p.value
**APC.TCA.levels**					
high.high	93 (46.3%)	19 (31.1%)	112 (42.7%)	0.461(0.238,0.877)	**0.02**
high.low	16 (8.0%)	7 (11.5%)	23 (8.8%)	0.988(0.35,2.568)	0.981
low.high	22 (10.9%)	4 (6.6%)	26 (9.9%)	0.411(0.113,1.183)	0.128
low.low	70 (34.8%)	31 (50.8%)	101 (38.5%)	ref	ref
**APC.PD_L1**					
high.high	86 (42.8%)	17 (27.9%)	103 (39.3%)	0.428(0.214,0.834)	**0.01**
high.low	23 (11.4%)	9 (14.8%)	32 (12.2%)	0.848(0.337,2.006)	0.714
low.high	27 (13.4%)	5 (8.2%)	32 (12.2%)	0.401(0.126,1.069)	0.088
low.low	65 (32.3%)	30 (49.2%)	95 (36.3%)	ref	ref
**APC.IDO1**					
high.high	77 (38.3%)	18 (29.5%)	95 (36.3%)	0.444(0.222,0.867)	**0.02**
high.low	32 (15.9%)	8 (13.1%)	40 (15.3%)	0.475(0.185,1.12)	0.102
low.high	35 (17.4%)	5 (8.2%)	40 (15.3%)	0.271(0.086,0.712)	**0.014**
low.low	57 (28.4%)	30 (49.2%)	87 (33.2%)	ref	ref
**TCA.PD_L1**					
high.high	93 (46.3%)	18 (29.5%)	111 (42.4%)	0.376(0.192,0.714)	**0**
high.low	22 (10.9%)	5 (8.2%)	27 (10.3%)	0.441(0.138,1.188)	0.129
low.high	20 (10.0%)	4 (6.6%)	24 (9.2%)	0.388(0.107,1.125)	0.107
low.low	66 (32.8%)	34 (55.7%)	100 (38.2%)	ref	ref
**TCA.IDO1**					
high.high	89 (44.3%)	18 (29.5%)	107 (40.8%)	0.386(0.197,0.738)	**0.01**
high.low	26 (12.9%)	5 (8.2%)	31 (11.8%)	0.367(0.116,0.976)	0.06
low.high	23 (11.4%)	5 (8.2%)	28 (10.7%)	0.415(0.13,1.116)	0.102
low.low	63 (31.3%)	33 (54.1%)	96 (36.6%)	ref	ref

Supplementary Table 3: Odds ratios (ORs) for 5-year recurrence events in FinXX and Mayo Clinic TNBC cohorts for intraepithelial APC eigenprotein scores in combination with TCA eigenprotein score, PD-L1, or IDO1 protein levels. Scores were sorted into two categories for each feature: high defined as > median or low defined as < median. Two-sided p-values for OR are given ($p<0.05$ in bold font). Eigenprotein scores are in Supplementary Data 5. Source Data are in Supplementary Data 2 (FinXX) and 7 (TNBC TMA).

FinXX Intraepithelial

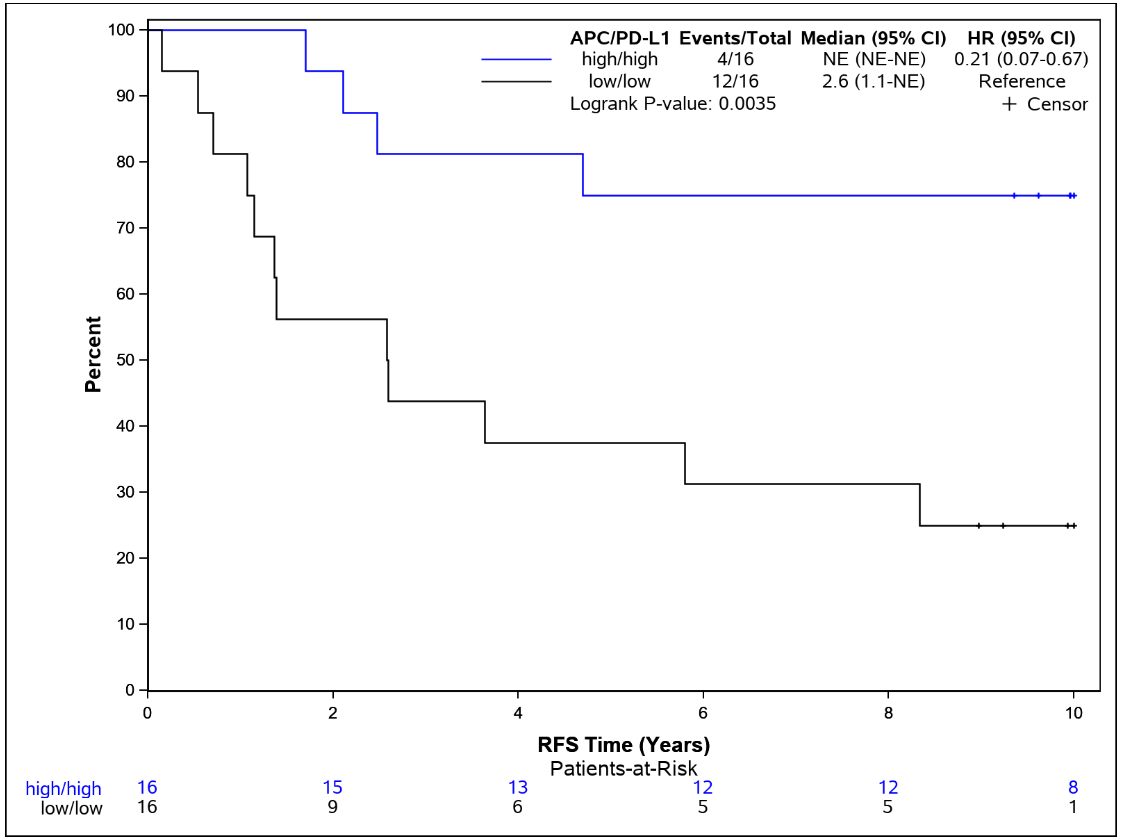

TNBC_TMA Intraepithelial

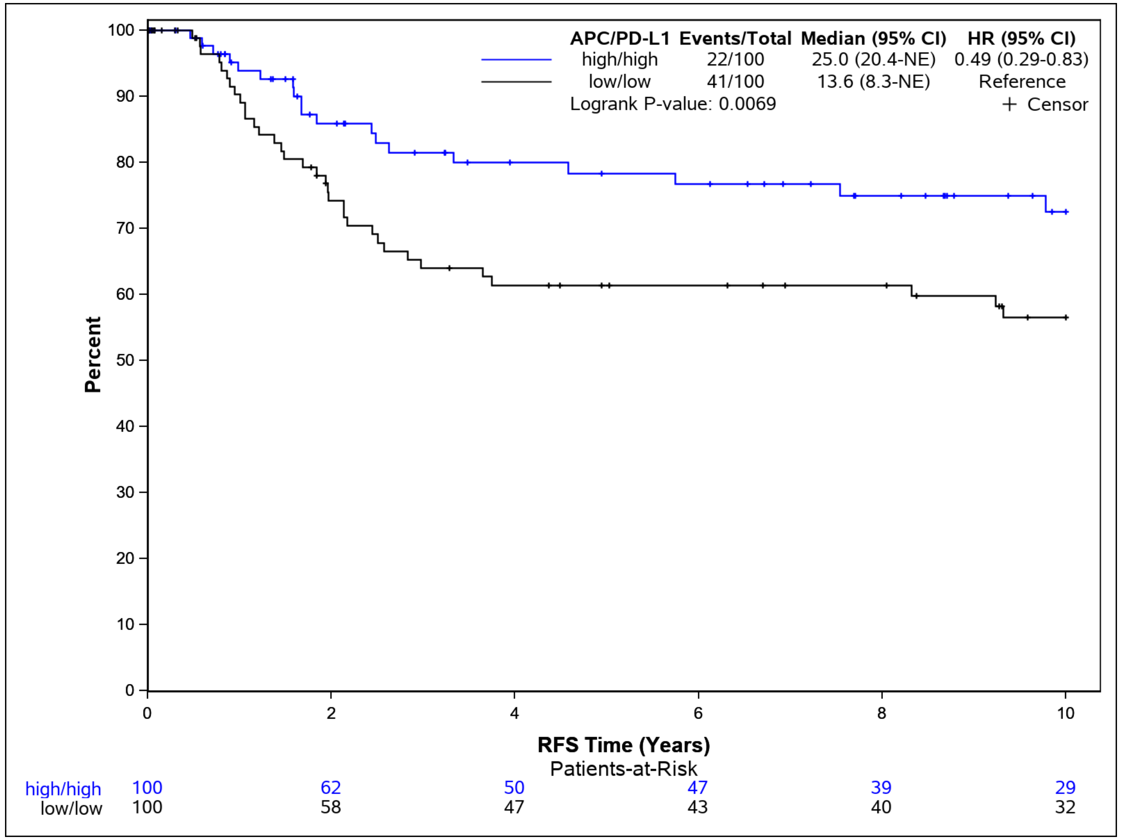

Supplementary Figure 17: Kaplan-Meier analysis comparing intraepithelial APC eigenprotein score >median and PD-L1 protein levels >median (high/high, blue) versus APC<median and PD-L1 <median (low/low, black) in FinXX (left) and Mayo Clinic TNBC TMA samples (right). CoxHRs, median survival, and log rank p-values are given in the insert. Source data for eigenprotein scores are in Supplementary Data 5 and for PD-L1 abundance in Supplementary Data 2 (FinXX) and 7 (TNBC_TMA).

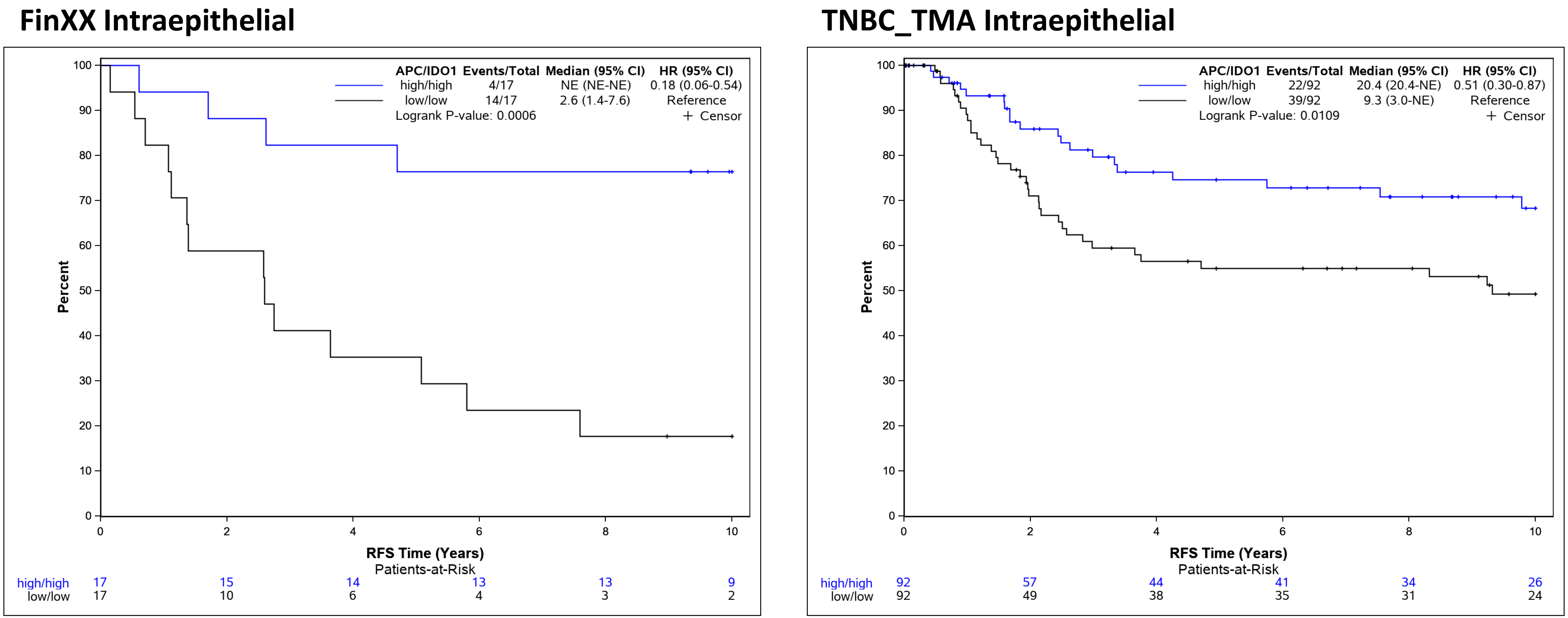

Supplementary Figure 18: Kaplan-Meier comparing high vs. low combined APC eigenprotein/IDO1 protein levels in FinXX (left) and Mayo Clinic TNBC TMA samples (right). For both features, high is defined as > median, and low is defined as < median with APC/IDO1 high/high (blue line) vs. APC/IDO1 low/low (black line). CoxHRs, median survival, and log rank p-values are given in the insert. Source data for eigenprotein scores are given in Supplementary Data 5. Source data for IDO1 abundance are in Supplementary Data 2 (FinXX) and 7 (TNBC_TMA).

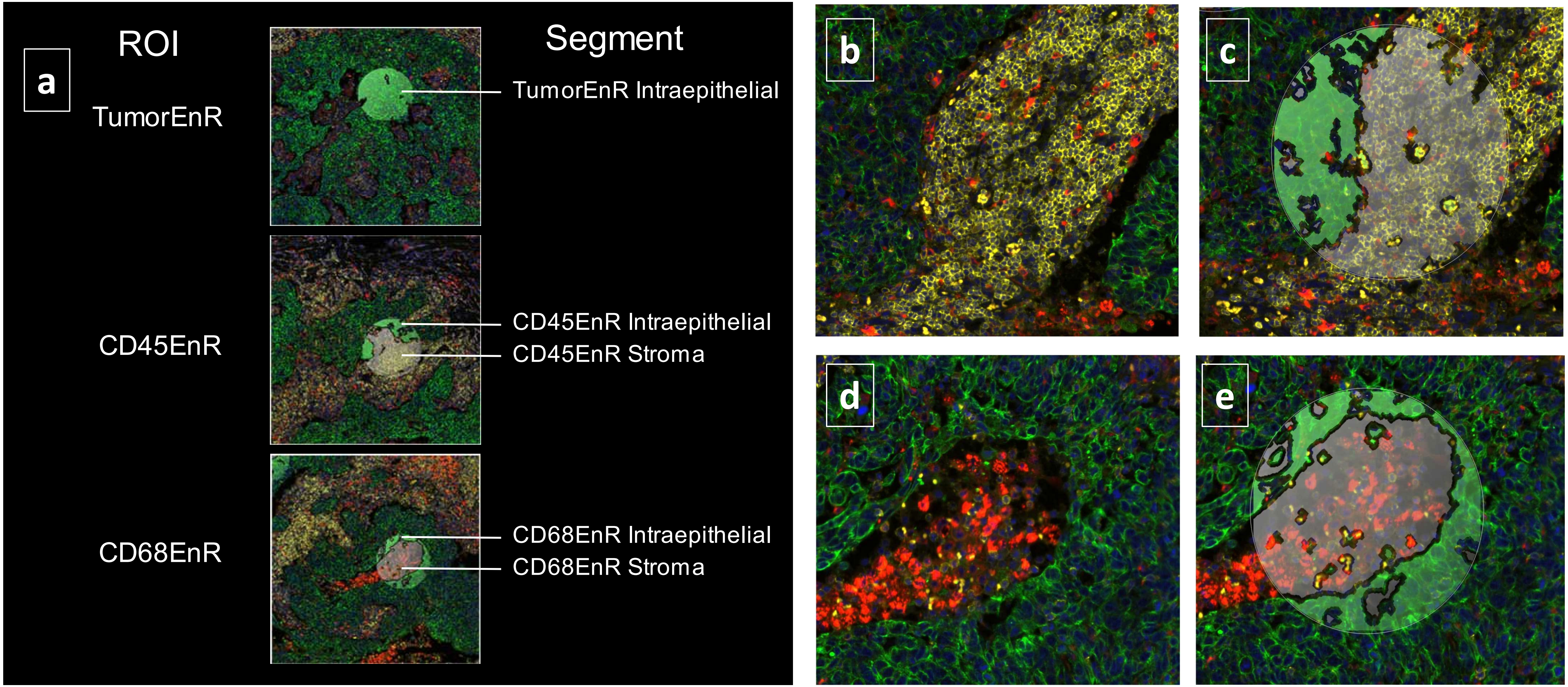

Supplementary Figure 19: ROI selection and Segmentation strategy in FinXX samples. 600 micron-diameter circular regions-of-interest (ROIs) were selected from tumor regions with little or no adjacent stroma (TumorEnR ROI in **Panel a**), regions with abundant tumor-nest adjacent stromal CD45-positive cells (CD45EnR in **Panel a**) or those with abundant CD68-positive stromal cells (CD68EnR in **Panel a**). High magnification **Panels b-e** illustrate segmentation of ROIs into Intraepithelial, cytokeratin-positive (light green) and stromal, cytokeratin-negative (gray) segments (areas of illumination). Green=Pan-cytokeratin, Red=CD68, Yellow=CD45 immunofluorescent labelling.
