# Supplementary material for: Distinct spatial immune microlandscapes are independently associated with outcomes in triple-negative breast cancer
Source: Nat Commun. 2023 Apr 18;14:2215. doi: 10.1038/s41467-023-37806-0 (PMC10113250; doi:10.1038/s41467-023-37806-0)
Supplement: Supplementary file 3 — Description of Additional Supplementary Files [file 41467_2023_37806_MOESM3_ESM.pdf]

**Description of Additional Supplementary Files**

**Supplementary Data 1: FinXX normalized protein counts for individual segments.** AOI counts in Source Data 1 were filtered for quality and normalized as described in Methods.

**Supplementary Data 2: FinXX normalized tumor average protein counts.** Segment level counts were averaged, as described in Methods. Source Data are given in Supplementary Data 1. Normalized tumor average counts were used for all outcome analyses.

**Supplementary Data 3: FinXX average counts for 29 proteins by segment.** Counts for each protein in each segment were averaged. Source Data are in Supplementary Data 2.

**Supplementary Data 4: TNBC TMA demographics.** This file contains clinical/pathological data for all TNBC TMA samples included in this analysis.

**Supplementary Data 5: Eigenprotein scores all samples.** Eigenprotein scores were calculated for FinXX and TNBC TMA samples. Source Data are Supplementary Data 1 (FinXX) and 7 (TNBC TMA).

**Supplementary Data 6: Normalized data for supplementary figures by fold change.** Fold change data were calculated using the linear mixed model from Source Data in Supplementary Data 1 (FinXX) and 7 (TNBC TMA).

**Supplementary Data 7: TNBC TMA segment level normalized counts.** Protein counts were normalized as described in Methods using data from Source Data 2.
