# Supplementary material for: Distinct spatial immune microlandscapes are independently associated with outcomes in triple-negative breast cancer
Source: Nat Commun. 2023 Apr 18;14:2215. doi: 10.1038/s41467-023-37806-0 (PMC10113250; doi:10.1038/s41467-023-37806-0)
Supplement: Supplementary file 11 — Reporting Summary [file 41467_2023_37806_MOESM11_ESM.pdf]

nature portfolio

Corresponding author(s): E. Aubrey Thompson

Last updated by author(s): Mar 28, 2023

Reporting Summary

Nature Portfolio wishes to improve the reproducibility of the work that we publish. This form provides structure for consistency and transparency in reporting. For further information on Nature Portfolio policies, see our Editorial Policies and the Editorial Policy Checklist.

Statistics

For all statistical analyses, confirm that the following items are present in the figure legend, table legend, main text, or Methods section.

n/a	Confirmed	
☐	☒	The exact sample size (*n*) for each experimental group/condition, given as a discrete number and unit of measurement
☐	☒	A statement on whether measurements were taken from distinct samples or whether the same sample was measured repeatedly
☐	☒	The statistical test(s) used AND whether they are one- or two-sided<br>*Only common tests should be described solely by name; describe more complex techniques in the Methods section.*
☐	☒	A description of all covariates tested
☐	☒	A description of any assumptions or corrections, such as tests of normality and adjustment for multiple comparisons
☐	☒	A full description of the statistical parameters including central tendency (e.g. means) or other basic estimates (e.g. regression coefficient) AND variation (e.g. standard deviation) or associated estimates of uncertainty (e.g. confidence intervals)
☐	☒	For null hypothesis testing, the test statistic (e.g. *F*, *t*, *r*) with confidence intervals, effect sizes, degrees of freedom and *P* value noted<br>*Give P values as exact values whenever suitable.*
☒	☐	For Bayesian analysis, information on the choice of priors and Markov chain Monte Carlo settings
☒	☐	For hierarchical and complex designs, identification of the appropriate level for tests and full reporting of outcomes
☒	☐	Estimates of effect sizes (e.g. Cohen's *d*, Pearson's *r*), indicating how they were calculated

Our web collection on statistics for biologists contains articles on many of the points above.

Software and code

Policy information about availability of computer code

Data collection	No software was used for data collection
Data analysis	All secondary analyses were done with R Statistical Software version 4.1.2. Quality control and normalization were carried out using a beta version of the GeoMx software for FinXX and version 1.3.0.20 for the TNBC TMA data.

For manuscripts utilizing custom algorithms or software that are central to the research but not yet described in published literature, software must be made available to editors and reviewers. We strongly encourage code deposition in a community repository (e.g. GitHub). See the Nature Portfolio guidelines for submitting code & software for further information.

Data

Policy information about availability of data

All manuscripts must include a data availability statement. This statement should provide the following information, where applicable:

- Accession codes, unique identifiers, or web links for publicly available datasets
- A description of any restrictions on data availability
- For clinical datasets or third party data, please ensure that the statement adheres to our policy

All data used in this study are available as a supplement to the manuscript (Supplementary Data). Source data for FinXX , normalized data, counts/segment for all ROIs and genes, are given in Supplementary Data 1. Source data for tumor average counts/segment are given in Supplementary Data 2. Source data for Mayo Clinic TNBCTMA are given in Supplementary Data 7. Source data for eigenprotein scores are given in Supplementary Data 5.

Human research participants

Policy information about studies involving human research participants and Sex and Gender in Research.

Reporting on sex and gender	Only female breast cancer patients were enrolled in the FinXX trial or included in the Mayo Clinic TNBC TMA cohort.
Population characteristics	Patient demographics for the FinXX cohort are given in Supplementary Table 1, and demographics for the Mayo Clinic TNBC TMA cohort are given in Supplementary Data 4.
Recruitment	No patients were recruited for these analyses.
Ethics oversight	This study was conducted in accordance with recognized ethical guidelines including the U.S. Common Rule. The FinXX study was approved by an Institutional Review Board at the Helsinki University Hospital (approvals 264/13/03/02/2014 and HUS/903/2017). The patients who participated in the FinXX trial (NCT00114816) signed a written informed consent to the trial participation and a consent to allow the use of their tumor tissue for FinXX trial-related research purposes. Analysis of Mayo Clinic TNBC samples was approved by the Mayo Clinic Institutional Review Board. Written informed consent was obtained from all patients for the use of tumor samples for research purposes.

Note that full information on the approval of the study protocol must also be provided in the manuscript.

Field-specific reporting

Please select the one below that is the best fit for your research. If you are not sure, read the appropriate sections before making your selection.

- [x] Life sciences
- [ ] Behavioural & social sciences
- [ ] Ecological, evolutionary & environmental sciences

For a reference copy of the document with all sections, see nature.com/documents/nr-reporting-summary-flat.pdf

Life sciences study design

All studies must disclose on these points even when the disclosure is negative.

Sample size	From the FinXX trial, a case/control design was used to identify proteins that were differentially associated with outcome. We selected all patients who had a recurrence (22 patients total, 11 from each treatment arm) and 22 patients without recurrence (11 from each arm), matched for patient age, tumor grade, tumor size and lymph node status for digital spatial profiling (DSP) of FFPE-derived tumor sections. Specifically, a total of 841 ROIs (from individual tissue cores) passed quality control for nuclear content and segment area: 416 intraepithelial segments and 425 stroma segments; corresponding to 275 unique tumors from the TNBC cohort with intraepithelial and stroma segment-level DSP data.
Data exclusions	no data exclusion
Replication	Two independent sample cohorts were analyzed.
Randomization	Not applicable
Blinding	Investigators were not blinded to clinical outcome.

Reporting for specific materials, systems and methods

We require information from authors about some types of materials, experimental systems and methods used in many studies. Here, indicate whether each material, system or method listed is relevant to your study. If you are not sure if a list item applies to your research, read the appropriate section before selecting a response.

Materials & experimental systems

n/a	Involved in the study	
[ ]	[x]	Antibodies
[x]	[ ]	Eukaryotic cell lines
[x]	[ ]	Palaeontology and archaeology
[x]	[ ]	Animals and other organisms
[x]	[ ]	Clinical data
[x]	[ ]	Dual use research of concern

Methods

n/a	Involved in the study	
[x]	[ ]	ChIP-seq
[x]	[ ]	Flow cytometry
[x]	[ ]	MRI-based neuroimaging

Antibodies

Antibodies used	Anti-CD68 (Novus NBP2-34736AF647, clone SPM130, 0.25ug/ul), labeled with Alexa Fluor 647 was obtained from NovusBio. Anti-pan-cytokeratin labeled with Alexa Fluor 532 (Novus NBP2-33200AF532, clones A1+A3, lot 42604602, 0.5ug/ul) and anti-CD45 labeled with Alexa Fluor 594 (Novus NBP2-34528AF594, clones 2B11 + PD7/26, lot 42604402, 5.0ug/ul) plus SYTO13 were obtained from NanoString.
Validation	Validation of the antibodies listed in Supplementary Data 1 is described in Merritt et al. 23.
